# An Improved Energy-Efficient Routing Protocol for Wireless Sensor Networks

**DOI:** 10.3390/s19204579

**Published:** 2019-10-21

**Authors:** Yang Liu, Qiong Wu, Ting Zhao, Yong Tie, Fengshan Bai, Minglu Jin

**Affiliations:** 1 College of Electronic Information Engineering, Inner Mongolia University, Hohhot 010021, China; yangliu@imu.edu.cn (Y.L.); tingzhao@mail.imu.edu.cn (T.Z.); eefs@imu.edu.cn (F.B.); 2 School of Biological Science and Medical Engineering, Beihang University, Beijing 100083, China; qiongwuimu@163.com; 3 Faculty of Electronic Information and Electrical Engineering, Dalian University of Technology, Dalian 116024, China; mljin@dlut.edu.cn

**Keywords:** wireless sensor networks, routing protocol, energy efficiency, network lifetime

## Abstract

Cluster-based hierarchical routing protocols play an essential role in decreasing the energy consumption of wireless sensor networks (WSNs). A low-energy adaptive clustering hierarchy (LEACH) has been proposed as an application-specific protocol architecture for WSNs. However, without considering the distribution of the cluster heads (CHs) in the rotation basis, the LEACH protocol will increase the energy consumption of the network. To improve the energy efficiency of the WSN, we propose a novel modified routing protocol in this paper. The newly proposed improved energy-efficient LEACH (IEE-LEACH) protocol considers the residual node energy and the average energy of the networks. To achieve satisfactory performance in terms of reducing the sensor energy consumption, the proposed IEE-LEACH accounts for the numbers of the optimal CHs and prohibits the nodes that are closer to the base station (BS) to join in the cluster formation. Furthermore, the proposed IEE-LEACH uses a new threshold for electing CHs among the sensor nodes, and employs single hop, multi-hop, and hybrid communications to further improve the energy efficiency of the networks. The simulation results demonstrate that, compared with some existing routing protocols, the proposed protocol substantially reduces the energy consumption of WSNs.

## 1. Introduction

Wireless sensor networks (WSNs) generally consist of considerable sensor nodes (SNs) with limited energy. WSNs are randomly deployed in a particular region to acquire various types of environmental parameters and transmit information to the base station (BS) for monitoring and detecting applications [1]. They have been widely applied in forest fire detection, surveillance, military, human health detection, etc., and thus have attracted the interest of researchers in recent years [2,3]. Because WSNs are usually deployed in hazardous environments, recharging or replacing the batteries of the SNs is very difficult. Moreover, the manual operation of the network is highly difficult, which brings some challenges regarding the application of WSNs [4,5,6]. To remedy these drawbacks, the efficient use of the battery energy of SNs should be considered as a primary goal when researchers design protocols and hardware architectures [7]. Therefore, several routing protocols have been proposed to render the sensor network more energy efficient [8,9].

The cluster formation and various communication modes of transmitting data have been the most emphasized approaches. In general, compared with non-clustering protocols, cluster-based routing protocols can efficiently use the SNs in the network [10]. A cluster leader, called the cluster head (CH), is in charge of eliminating the correlated data that can decrease the final data volume. Afterwards, the CH will transmit the aggregated data to the BS [11,12,13]. In cluster-based routing protocols, SNs are divided into many clusters to decrease energy consumption for long distance communication. The clustering can minimize the overall energy consumption and balance the nodes’ workload, which is caused by the large difference in the energy depletion between the CHs and other nodes. Therefore, clustering is an energy-efficient solution for increasing network longevity and improving energy efficiency. Moreover, most clustering protocols adopt optimal CH selection to avoid the premature death of the SNs and further extend the lifetime of the network [14,15,16].

According to [17], we know that SNs consume more energy during communication than during the computation process. In contrast, some other protocols adopt multi-hop communication, and the nodes close to the BS have excessive transmission overhead, leading to energy holes in the sensor field [18]. To address the energy hole problem and prolong the network lifetime, many clustering protocols have been specifically proposed for WSNs. The low-energy adaptive clustering hierarchy (LEACH) is the most important hierarchical routing protocol in terms of saving energy compared with traditional routing protocols [19]. In this type of protocol, the entire network is divided into several clusters, and each cluster selects a node in a probabilistic manner as a CH that is in charge of receiving, aggregating, compressing, and sending the information collected from other non-CH nodes to the BS. With the objective of minimizing the energy consumption of the WSNs, the LEACH routing protocol designates a single CH node in each cluster and selects the CH on a rotation basis. Although the efficiency of the LEACH protocol has been closely studied, it still has some drawbacks that require improvement—on one hand because selecting the CH is based on a random round robin, the number of CHs is unreasonable in each round, and the nodes at the boundary of the network will be elected as the CH. On the other hand, there is no consideration of the distribution of the CHs, the threshold condition, and the remaining energy of each node after the end of each round. Consequently, these issues will result in energy overheads.

To remedy this problem, based on this classical hierarchical clustering protocol, a variety of protocols have been proposed [19]. In contrast to the LEACH protocol, a novel distributed protocol called the scalable energy-efficient clustering hierarchy (SEECH) assigns CHs and relays roles to different nodes [20]. This protocol generates two fitness functions to determine the capability to become a CH or relay for each node by only taking their energy consumption into account. However, in the SEECH protocol, every node may be elected to be a CH or a relay, including the nodes closer to the BS, which increases the energy consumption. A learning automata-based multilevel heterogeneous routing (LA-MHR) scheme was proposed in [21]. The LA-MHR utilizes S-model-based learning automata for selecting CHs, while the cognitive radio spectrum is allocated by the BS. In addition, single hop communication among different SNs is replaced by a multi-hop communication approach in the LA-MHR scheme. Since a single communication approach is not suitable for scenes in which the CHs are far from the SNs, this approach requires a large amount of transmission power. The LEACH-Mobile (LEACH-M) protocol is proposed for mobile nodes to improve data transfer success rate [22]; however, the cluster head selection and cluster formation for LEACH-M are similar to the LEACH protocol. Because of the high control overheads, the energy dissipation of LEACH-M is greater than LEACH. The optical LEACH (O-LEACH) protocol divides and links a series of WSNs that is around the distributed fiber sensors (DFS) in the hybrid sensor network with a rectangular topology [23]. Compared with LEACH, this method extends the lifetime of WSNs and exhibits good energy efficiency. However, the energy consumption of O-LEACH is not improved. The stable energy efficient network (SEEN) has been proposed for WSNs [24]. The selection of Cluster Head in this protocol is done on the basis of the residual energy of nodes as well as the distance parameter. In wireless sensor networks, few sensor nodes are equipped with higher processing and communicating capabilities. In [25], an energy consumption-minimizing strategy called LEACH-XMP (LEACH-eXtended Message-Passing) was proposed, which adopts a sophisticated energy consumption model for CH nodes without oversimplification. These protocols aim to merely distribute the energy consumption between the CH and its assistant at the beginning of the first round. This distribution will result in a reduction in the network lifetime.

To overcome the drawbacks of conventional methods and further prolong the lifetime of WSNs, we propose a novel improved energy-efficient LEACH (IEE-LEACH) routing protocol in this paper. In the proposed protocol, the threshold setting introduces four parameters including the initial energy of nodes, residual energy of nodes, total energy of the network, and average energy of the network. In the proposed IEE-LEACH protocol, the node closer to the BS than the CH does not take part in the cluster formation. Thus, the protocol can balance the energy load and decrease the energy consumption. Furthermore, the proposed IEE-LEACH protocol compares the energy consumption of single hop and multi-hop communication modes in the data transmission phase. The communication mode with the least energy consumption will be adopted. Therefore, the proposed approach decreases the overall communication cost and significantly improves the network lifetime.

The remainder of this paper is organized as follows. Section 2 briefly introduces the system model. The proposed IEE-LEACH protocol is indicated in Section 3. We demonstrate the performance evaluation of the proposed protocol via simulations compared to the conventional protocols in Section 4. Finally, conclusions are drawn in Section 5.

## 2. System Model

### 2.1. Network Model and Assumptions

In this section, the sensor network is introduced as the WSN model. The *n* SNs are uniformly and randomly placed in L∗L square area and are manually deployed in a complex environment. The set of SNs is given by

(1) $$S=(s_{1},s_{2},\ldots \ldots s_{n}).$$

**Figure 1:**
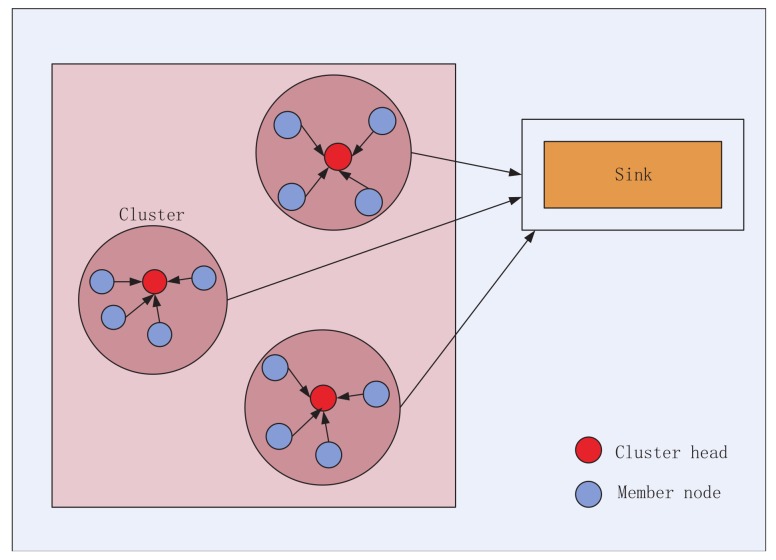
Network structure.

The structure of the network is illustrated in Figure 1. Each cluster has its own CH and cluster members. Each cluster member collects the data and forwards it to the CH. Then, all CHs with the function of aggregating and compressing the information will process the data and then transmit the information to the sink node.

During the deployment of the nodes, the underlying network model and the deployment of SNs are assumed as follows:

(1) A sink node that is relatively far from the WSN is powered by manually, without considering the energy consumption issue.

(2) The SNs and sink node are immobile.

(3) The SNs are set with the same initial energy, and each SN is assigned a unique identification in the network.

(4) The SNs have the ability to adjust the wireless transmission power on the basis of a specific situation.

(5) The node senses the information of the surrounding environment and is in the state of sending data all the time.

### 2.2. Energy Consumption Model

**Figure 2:**
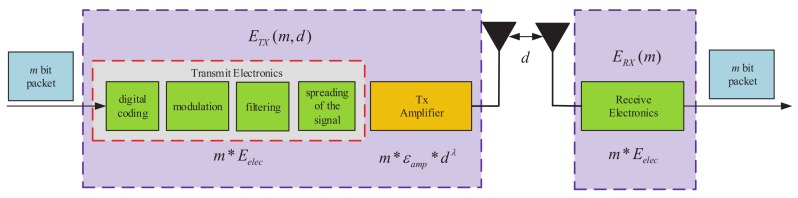
Radio energy dissipation model.

The radio hardware energy consumption model is shown in Figure 2. The radio electronics and the power amplifier are run by the transmitter, and the radio electronics are also run by the receiver. Both operations consume energy. The energy consumption model adopts the free space channel with $d^{2}$ energy consumption and the multipath channel with $d^{4}$ energy consumption depending on the distance between the transmitter and the receiver [16,17,18,19,20]. Thus, to transmit a packet of *m* bits at a distance *d*, the energy consumption is given by the following equation:

(2) $$E_{TX}=\left\{\begin{array}{cc} m*E_{elec}+m*\varepsilon_{fs}*d^{2} & d\leq d_{0},\\ m*E_{elec}+m*\varepsilon_{mp}*d^{4} & d>d_{0}, \end{array}\right.$$

where $d_{0}$ can be calculated by d0=εfsεfsεmpεmp. The electronic energy $E_{elec}$ is used depending on the digital coding, modulation, filtering, and spreading of the signal. The specific energy function of receiver is the reverse of the transmitter, as depicted in Figure 2. The parameters $\varepsilon_{fs}$ and $\varepsilon_{mp}$ are the amplification factors of the transmitting circuit when $d\leq d_{0}$ and $d>d_{0}$, where $d_{0}$ is threshold. To receive a message of *m* bits, the receiver expends energy as follows:

(3) $$E_{RX}=m*E_{elec}.$$

## 3. Proposed Scheme

### 3.1. LEACH Protocol

**Figure 3:**
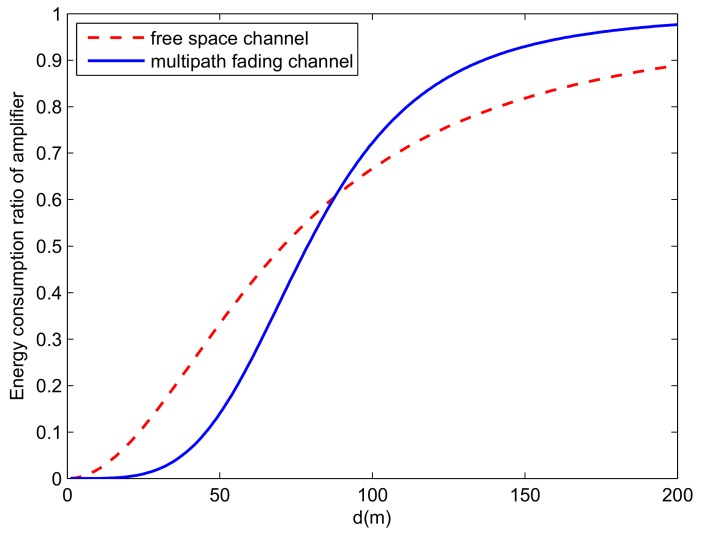
The power amplifier energy consumption to total energy consumption ratio.

The LEACH protocol plays an important role in the field of WSNs [26,27]. In the CH election phase, the CH of every cluster compresses the data collected from the member nodes and then sends them to the sink node [9]. The mechanism of selecting the CH is determined by the threshold value T(n) and the rand function. The SN generates a random number *M*(0≤M<1). If it is satisfied with M≤T(n), the node is elected as the head node of the cluster for the current round. The limit value T(n) is calculated as follows:

(4) $$T\left(n\right)=\left\{\begin{array}{cc} \frac{p}{1-p*(r*\;\mathrm{mod}\;(1/p))}, & n\in G,\\ 0, & \mathrm{otherwise}, \end{array}\right.$$

where *p* is the ratio of the total number of CHs to SNs and represents the probability of each node becoming CH during round 0 [4], *r* is the current number of rounds, *G* is the set of the nodes that will not be elected as a CH in a recent 1/p round, and mod(·) denotes the modulus operator. The power amplifier energy consumption to total energy consumption ratio versus the distance is shown in Figure 3. The power amplifier energy consumption to total energy consumption ratio can be calculated by:

(5) $$ratio=\left\{\begin{array}{c} \frac{m*\varepsilon_{fs}*d^{2}}{m*E_{elec}+m*\varepsilon_{fs}*d^{2}},d\leq d_{0},\\ \frac{m*\varepsilon_{mp}*d^{4}}{m*E_{elec}+m*\varepsilon_{mp}*d^{4}},d>d_{0}. \end{array}\right.$$

The LEACH protocol adopts the concept of clustering and periodic data collection, which can reduce the data transmission between the nodes and the BS. Therefore, this protocol can not only reduce the energy loss, but also can extend the network lifetime. In addition, the CH uses the method of data aggregation, which can reduce correlated data locally. This method can also optimize the amount of data in the network and reduce energy consumption. Moreover, the time division multiple access (TDMA) schedule used by LEACH allows the member nodes to go into sleep mode, and this mechanism holds back the collision between clusters and extends the sensors’ battery life [28,29,30].

However, the density of nodes is not considered in the traditional LEACH protocol when selecting the CH. The placement of nodes and the expected number of CHs per round are considered when assigning CHs. Therefore, this protocol cannot ensure the uniform distribution of the CHs [31]. Additionally, the LEACH protocol does not consider the residual energy of nodes and the average energy of all nodes when selecting the CH. This will lead to a node with a lower energy being chosen as the CH. Thus, this protocol leads to the quick exhaustion of the node energy [31]. Finally, the CH communicates directly with the BS by adopting a single hop communication mode. If the BS is far from some of the CH nodes, about 80% of the energy consumption of the node comes from the power loss of the long-distance data transmission [4]. Under the free space channel model, the power amplifier energy consumption to total energy consumption ratio is about 80% when d≈141m. In addition, under the multipath fading channel model, the power amplifier energy consumption to total energy consumption ratio is about 80% when d≈112m. Therefore, looking towards real applications, it is necessary to develop an energy-efficient protocol to decrease the energy loss of the WSN.

### 3.2. Improved Energy-Efficient LEACH Protocol

To decrease the energy loss and increase the energy efficiency of the WSNs, we propose a novel improved energy-efficient LEACH protocol called IEE-LEACH in this section.

#### 3.2.1. Energy Consumption Model of the IEE-LEACH Protocol

The energy consumption model of the proposed IEE-LEACH protocol is introduced in this section. Unlike the LEACH protocol, in which nodes with the same initial energy are considered, each SN in the IEE-LEACH protocol is assigned a different initial energy. It is assumed that there are *N* nodes that are placed in an L×L region, and the proposed IEE-LEACH protocol takes two models into accounts, i.e., the free space model and multipath model.

When the BS is close to the nodes, the energy dissipated follows the free space model ($d^{2}$ power loss) [17]. Thus, the energy consumption of the CH per round is

(6) $$\begin{array}{cc} E_{CH}= & \left(\frac{N}{k}-1\right)*m*E_{elec}+\frac{N}{k}*m*E_{DA}\\ \; & +m*E_{elec}+m*\varepsilon_{fs}*d_{toBS}^{2}, \end{array}$$

where *k* is the number of clusters per round in the WSN, N/k is the average node of each cluster, $E_{DA}$ is the energy consumption of the CH receiving a message of 1 bit, and $d_{toBS}^{2}$ denotes the expected squared distance between the CH and BS [12]. Without the loss of generality, the node ρ(x,y) is distributed in an arbitrary-shaped region. It is assumed that the base station is located at location (a,b). The expected squared distance between the CH and BS can be expressed as

(7) $$\begin{array}{cc} E[d_{toBS}^{2}] & =\int \int ({\left(a-x\right)}^{2}+{\left(b-y\right)}^{2})\rho \left(x,y\right)dxdy\\ & =\int \int \frac{{\left(a-x\right)}^{2}+{\left(b-y\right)}^{2}}{A}dxdy, \end{array}$$

where *A* is the region of distributed nodes. The energy consumption of cluster members per round is

(8) $$E_{nonCH}=m*E_{elec}+m*\varepsilon_{fs}*d_{toCH}^{2},$$

where $d_{toCH}^{2}$ denotes the expected squared distance between one sensor and CH, which can be expressed as

(9) $$E\left[d_{toCH}^{2}\right]=\int \int \left(x^{2}+y^{2}\right)\rho \left(x,y\right)dxdy=\frac{L^{2}}{2\pi k}.$$

The energy consumption of each cluster is given by [12]

(10) $$\begin{array}{cc} E_{cluster} & =E_{CH}+\left(\frac{N}{k}-1\right)E_{nonCH}\\ \; & \approx E_{CH}+\frac{N}{k}E_{nonCH}. \end{array}$$

Thus, the energy consumption with the IEE-LEACH protocol in the WSN during each round is given by

(11) $$\begin{array}{cc} E_{round}= & kE_{cluster}\\ = & m(2NE_{elec}+NE_{DA}+k\varepsilon_{fs}d_{toBS}^{2}\\ & \;+N\varepsilon_{fs}d_{toCH}^{2}). \end{array}$$

The derivative of $E_{round}$ with respect to *k* is equal to the optimal number of CHs, which can be expressed as

(12) $$k_{opt}=\frac{\sqrt{N}}{\sqrt{2\pi }}\frac{L}{d_{toBS}}.$$

In addition, the energy dissipated follows the multipath model ($d^{4}$ power loss) when the BS is far from the nodes [21]. The energy consumption of the CH node is given by

(13) $$\begin{array}{cc} E_{CH}= & \left(\frac{N}{k}-1\right)*m*E_{elec}+\frac{N}{k}*m*E_{DA}\\ \; & +m*E_{elec}+m*\varepsilon_{mp}*d_{toBS}^{4}. \end{array}$$

The expected value of distance can be expressed as

(14) $$\begin{array}{cc} E[d_{toBS}^{4}] & =\int {\int \left({\left(a-x\right)}^{2}+{\left(b-y\right)}^{2}\right)}^{2}\rho \left(x,y\right)dxdy\\ & =\int \int \frac{{\left({\left(a-x\right)}^{2}+{\left(b-y\right)}^{2}\right)}^{2}}{A}dxdy. \end{array}$$

In the multipath fading channel model, the $E_{nonCH}$, $d_{toCH}^{2}$ and $E_{cluster}$ are the same as the free space channel model mentioned above. Thus, the energy consumption of the IEE-LEACH protocol in the WSN during each round can be expressed as

(15) $$\begin{array}{cc} E_{round}= & kE_{cluster}\\ = & m(2NE_{elec}+NE_{DA}+k\varepsilon_{mp}d_{toBS}^{4}\\ & \;+N\varepsilon_{fs}d_{toCH}^{2}). \end{array}$$

The derivative of $E_{round}$ with respect to *k* is equal to the optimal number of CHs, which is given by [32]

(16) $$k_{opt}=\frac{\sqrt{N}}{\sqrt{2\pi }}\sqrt{\frac{\varepsilon_{fs}}{\varepsilon_{mp}}}\frac{L}{d_{toBS}^{2}}.$$

#### 3.2.2. Cluster Head Selection Algorithm of the IEE-LEACH Protocol

Although there are advantages of using the LEACH protocol, it cannot guarantee the current residual energy of the CH. The selection of the threshold T(n) used by conventional LEACH-based protocols only consider whether the nodes will be selected to be the CH, without considering the node’s energy. Therefore, the CHs are randomly selected, and, if a node with less energy is chosen as CH, then they will quickly die. To balance the energy consumption and prolong the network lifetime, we propose a new threshold $T(s_{i})$, which is defined by

(17) $$T\left(s_{i}\right)=\left\{\begin{array}{cc} \frac{p_{i}}{1-p_{i}\left(r\;\mathrm{mod}\;\left(1/p_{i}\right)\right)}, & s_{i}\in G,\\ 0, & \mathrm{otherwise}, \end{array}\right.$$

where $s_{i}$ is the node and i∈[1,N]. The energy adjustment parameter $p_{i}$ is given by

(18) $$p_{i}=\frac{p*s_{i}*E_{r}^{i}*E_{i}}{E_{t}*E_{a}},$$

where *p* is the proportion of selecting the optimal CH, $E_{r}^{i}$ is the current residual energy of the *i*th node, $E_{i}$ is the initial energy of the *i*th node, $E_{t}$ is the total energy of the whole network, and $E_{a}$ is the average energy of all SNs in the WSN. It can be seen from Equation (18) that the initial energy of nodes, residual energy of nodes, total energy of the network and average energy of all nodes are all used to calculate the energy adjustment parameter. This improvement can ensure that each node dies at approximately the same time. Thus, the proposed protocol can balance the distribution of the energy load among nodes and prolong the network lifetime. If the residual energy of the nodes is more than $E_{a}$, then an improved threshold value $T(s_{i})$ can increase the possibility of selecting them as the CH. After IEE-LEACH operates *r* rounds, the average energy $E_{a}$ of all nodes is obtained

(19) $$E_{a}=\frac{E_{t}\left(1-\frac{r}{r_{\max}}\right)}{s_{i}}.$$

Initially, the nodes are randomly distributed in the WSN when the process of selecting the CH begins to be executed. Each node generates a random number, which is compared with $T(s_{i})$. If the result is less than or equal to $T(s_{i})$, then the node will become the CH.

The newly proposed algorithm can increase the probability that the node whose residual energy is higher than the energy of the neighboring node or the average energy of the whole network becomes the CH node in the current round. Notably, all nodes have different energies with the IEE-LEACH protocol when selecting the CH. This process can ensure that each node dies at approximately the same time by more frequently selecting nodes with more residual energy than nodes with less residual energy as the CHs. Therefore, the IEE-LEACH protocol can lead to an increase in the lifetime of the overall network.

#### 3.2.3. Cluster Formation Algorithm of the Proposed IEE-LEACH

Once the election of CHs is completed, the CHs will inform the other nodes of the information that they have become the CH in this round. To complete this function, each CH node will send an advertisement message to all the other nodes in the form of broadcast with a non-persistent carrier-sense multiple access (CSMA) MAC protocol [33].

Each member node decides whether to participate in cluster formation, according to the signal intensity of the message that is transmitted from the BS and each CH node. Since the symmetric propagation channel model is used by the proposed IEE-LEACH protocol, the election of a cluster head is only related to the pure signal strength [15]. The stronger the received signal is, the closer the distance between member nodes to the sink node or the CH is. Comparing the distance of the nodes to the CHs and that of the nodes to the BS, the nodes closer to the base station do not participate in cluster formation and directly send data information to the BS.

**Figure 4:**
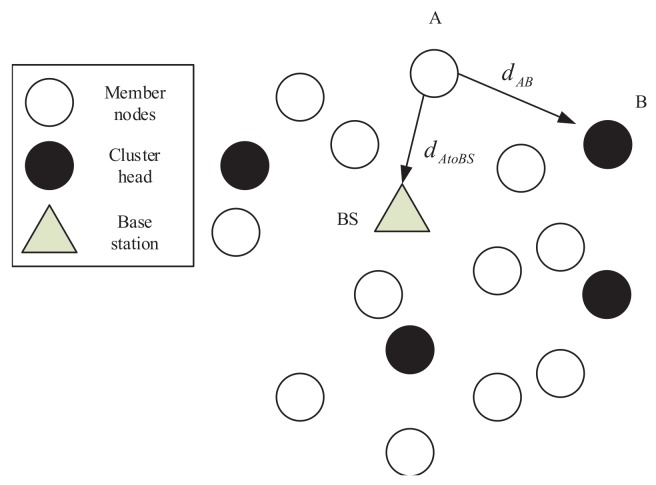
Cluster formation.

As shown in Figure 4, when the cluster head in the network is elected and the cluster head broadcasts messages to other nodes, the non-cluster head node A finds the nearest cluster head B according to the received signal strength, and calculates the distance between them, i.e., $d_{AB}$. When $d_{AB}>d_{AtoBS}$ ($d_{AtoBS}$ denotes the distance between the node A and BS), node A does not join any cluster and communicates directly with the base station during the data communication. When $d_{AB}<d_{AtoBS}$, node A joins the cluster where cluster head B is located and acts as a member node in the cluster.

This means that not all nodes in the WSN participate in cluster formation. A node closer to the CH decides to join the cluster whose CH transmits the strongest signal to the nodes by comparing all the signal intensities of the messages sent by the CHs. After each node closer to the CH determines which CH will join, it needs to inform the CH that it has become a member node of this cluster. Each node closer to the CH sends a join-request message (Join-REQ) to the chosen CH, which includes the node’s identification and the CH’s identification.

**Figure 5:**
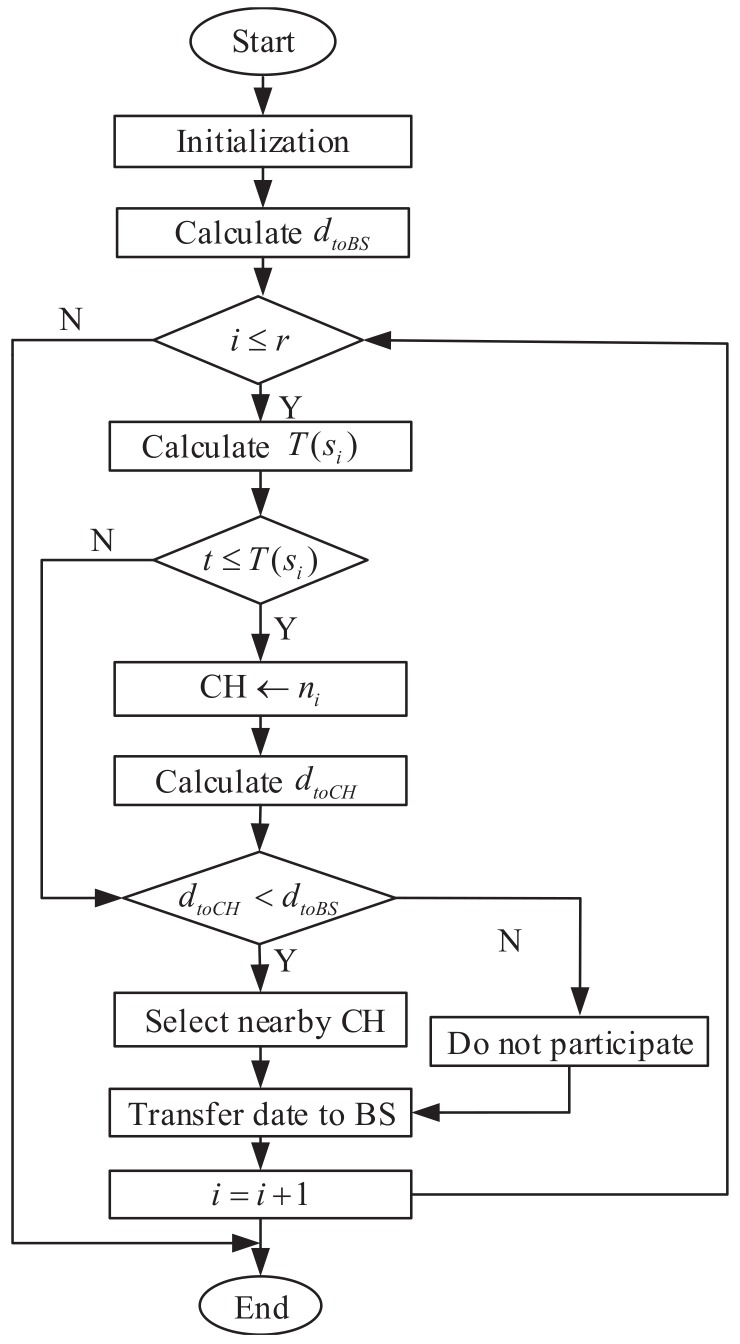
Block diagram of proposed protocol.

Figure 5 shows the process of CH selection and cluster formation of the proposed IEE-LEACH protocol. First, the related energy and the distance to the BS are calculated. Then, the threshold $T(s_{i})$ in each round is determined by Equation (17). Each node closer to the CH needs to generate a random number and then compare it with $T(s_{i})$. If the result is less than or equal to $T(s_{i})$, then the node will be chosen as the CH. The distance to CH is calculated. The nodes whose distance is closer to the BS rather than the CH will not take part in the process of cluster formation and will directly transmit data to the BS. Finally, the transmitted data adopt the method of single hop, multi-hop and hybrid communications.

#### 3.2.4. Data Transmission Scheme of the IEE-LEACH Protocol

To decrease energy consumption, the radio network of the CH and nodes is closed until the transmission slot is assigned. The radio network of the CH and nodes that intend to send the data need to be activated. After activating the nodes, non-CH nodes send the data to the CH, while the CH receives these data and sends them to the BS [34,35]. A single data communication approach will increase the energy consumption of the nodes because the best way to transfer data are to minimize the distance between nodes. Therefore, we propose a methodology that provides a single hop, multi-hop and hybrid communication network to minimize the distance.

It is assumed that the cluster head node transmits data to BS by (n−1) hops and the distance of each hop is *r* in the wireless sensor network with the free-space channel model. Therefore, to transmit an *m*-bit message, the energy consumption for signal hop is given by

(20) $$\begin{array}{cc} E_{1} & =E_{TX}\left(m,n\times r\right)\\ & =m\times E_{elec}+m\times \varepsilon_{fs}\times {\left(nr\right)}^{2}\\ & =m\times \left(E_{elec}+\varepsilon_{fs}\times n^{2}\times r^{2}\right). \end{array}$$

The energy consumption for multi-hop can be expressed as

(21) $$\begin{array}{cc} E_{2} & =n\times E_{TX}\left(m,r\right)+\left(n-1\right)\times E_{RX}\left(m\right)\\ \; & =n\times m\times \left(E_{elec}+\varepsilon_{fs}r^{2}\right)+\left(n-1\right)\times E_{elec}\times m\\ \; & =m\times \left[\left(2n-1\right)E_{elec}+\varepsilon_{fs}\times n\times r^{2}\right]. \end{array}$$

Comparing the energy consumption of two communication modes, if the energy consumption of a multi-hop is less than that of a single hop, i.e.,

(22) $$\begin{array}{c} m\times \left[\left(2n-1\right)E_{elec}+\varepsilon_{fs}\times n\times r^{2}\right]\\ <m\times \left(E_{elec}+\varepsilon_{fs}\times n^{2}\times r^{2}\right). \end{array}$$

Then, it can be devised as

(23) $$\begin{array}{c} r>\sqrt{\frac{2E_{elec}}{n\varepsilon_{fs}}}. \end{array}$$

Note that $E_{elec}=50\;\mathrm{nJ}/\mathrm{bit}$ and $\varepsilon_{fs}=10\;\mathrm{pJ}/\mathrm{bit}/m^{2}$, when n=2, it can be found that r>70m. Therefore, the energy consumption of multi-hop communication is less than that of single hop communication when r>70m. This mechanism is beneficial to the average energy dissipation of the network and prolongs the lifetime of network.

The Algorithm 1 of the proposed protocol is shown below:
**Algorithm 1. IEE-LEACH**1. *N*-number of nodes, *r*-number of rounds.2. The first step is to initialize the network parameters for WSN.3. The second step is the random deployment of the nodes in the network.4. The third step is the deployment of the BS in the network.5. Calculate $d_{toBS}$6. for i=1 to *r*7. $E_{a}=E_{t}\left(1-\frac{r}{r_{\max}}\right)/n$8. $p_{i}=p*n*E_{r}^{i}*E_{i}/\left(E_{t}*E_{a}\right)$9. $T\left(s_{i}\right)=p_{i}/\left(1-p_{i}\left(r\;\mathrm{mod}\;\left(1/p_{i}\right)\right)\right)$10. t=Random number11. If $\left(t\leq T\left(s_{i}\right)\right)$12. CH $\leftarrow n_{i}$13. Calculate $d_{toCH}$14. end if15. if $\left(d_{toCH}<d_{toBS}\right)$16. Select CH and join the cluster;17. else18. Nodes do not participate in the cluster19. end if20. Transfer to BS21. i=i+122. go to step 623. end

The conventional LEACH-based protocols do not consider the energy adjustment parameter, which exhausts the node energy quickly. However, the proposed IEE-LEACH protocol introduces four energy parameters when selecting the threshold, i.e., initial energy of nodes, residual energy of nodes, total energy of the network, and average energy of all nodes. This mechanism can balance the energy distribution of all nodes and improve energy efficiency. In addition, to decrease the energy consumption, the node closer to the BS than the CH will not participate in the cluster formation. Therefore, the robustness of the network is improved and the lifetime of the network is increased. Furthermore, the proposed protocol compares the energy consumption of single hop and multi-hop communication modes in data transmission. Then, the communication mode with less energy consumption will be adopted. Since the distance of a single hop is less than 70m as mentioned above, the single hop will be adopted when the distance between CH and BS is less than 70m. Otherwise, the multi-hop method will be adopted. This improvement is beneficial for the average energy dissipation that enhances the lifetime of the sensor network. Thus, the proposed IEE-LEACH protocol shows flexibility over conventional LEACH protocols in terms of selecting cluster heads, cluster formation, and the data transmission.

## 4. Simulation Results

In this section, to evaluate the performance of the proposed IEE-LEACH protocol in the WSN, the existing LEACH, LEACH-Centralized (LEACH-C) [18], LEACH-M [22], O-LEACH [23], energy-efficient LEACH (EE-LEACH) [36], Stable Energy Efficient Network (SEEN) [24], and LEACH-eXtended Message-Passing (LEACH-XMP) [25] protocols are used in comparison in the simulation experiments. Simulation experiments were carried out using MATLAB 2014a (MathWorks, Natick, MA, USA). A wireless sensor network system model consisting of 100 homogeneous SNs [37] randomly distributed in 100m×100m square area with different initial energies is considered. The BS is placed at the coordinate (50, 100). Therefore, the maximum distance between the SN and the BS is $50\sqrt{5}m$ (approximately 111.8 m). We run the simulation for 3500 rounds, and the value of the time interval between rounds is 20 s [4]. Table 1 shows the parameter configurations in the WSN that are employed in the simulation.

The performance metrics of the WSN that are used to evaluate the performance of the algorithm as follows [38,39]:

- Stability Period: The time span from the beginning of network operation until the first dead sensor occurs, which can be calculated by
  (24) $$T_{stability}=t_{FND}-t_{start},$$
  where $t_{FND}=20\times FND$, FND is the number of rounds after which the first sensor died, $t_{FND}$ is the time that the first dead sensor occurred, and $t_{start}$ is the time of the beginning of network operation.
- Network Lifetime: The time span from the beginning of network operation until the last live SN dies, which can be calculated by
  (25) $$T_{lifetime}=t_{LDN}-t_{start},$$
  where $t_{LND}=20\times LND$,LND is the number of rounds after which all sensor nodes died, and $t_{LND}$ is the time at which all sensor nodes died.
- The Amount of Transmitted Data: The total quantity of data sent from the nodes (including the CHs and other nodes) in the WSN to the BS can be calculated by
  (26) $$data=data_{CHtoBS}+data_{NtoBS},$$
  where $data_{CHtoBS}$ is the amount of transmitted data by the CHs to the BS, and $data_{NtoBS}$ is the amount of transmitted data by nodes to BS. In particular, in this paper, $data_{NtoBS}$ concerns nodes near the station of the base, and which does not contribute to the formation of clusters.
- Energy Consumption of Network: The energy consumption of wireless sensor networks mainly includes circuit energy consumption and power amplifier energy consumption, and the latter is dominant. The energy consumption of each round in the network can be calculated by Equation (11) or Equation (15) in different channel models.
- Number of CHs per Round: The number of nodes that send information aggregated from their cluster members directly to the BS is elected according to the threshold condition $T(s_{i})$. This can be calculated by Equation (12) or Equation (16) in different channel models.

In terms of the reliability and lifetime of the WSN, the stability period of the WSN is of great value to research because the network is mainly responsible for data transmission. Furthermore, more attention is paid to the lifetime of the WSN, the number of data transmissions (to the BS), and the residual node energy, which objectively reflects the network lifetime situation.

### 4.1. Network Lifetime and Stability Period

**Figure 6:**
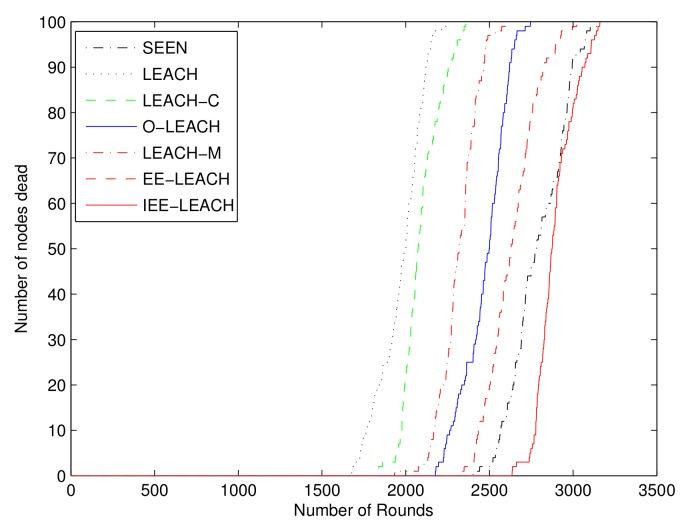
Comparison of life cycle.

This simulation illustrates the network lifetime of the proposed IEE-LEACH protocol compared with the other existing protocols. The number of dead nodes versus the number of rounds in the WSNs is shown in Figure 6. It is obvious that the nodes start to die after 1678 rounds, 1933 rounds, 2178 rounds, 1836 rounds, 2326 rounds, and 2416 rounds for LEACH, LEACH-M, O-LEACH, LEACH-C, EE-LEACH, and SEEN protocols, respectively. However, for the proposed IEE-LEACH, the nodes do not start to die until 2636 rounds. In addition, the number of rounds for all the nodes dead in the IEE-LEACH protocol is 3160 rounds as compared to 2245 rounds in the LEACH protocol. From the obtained results, the IEE-LEACH improves the network lifetime by approximately 9% to 57% compared with the other protocols.

### 4.2. Residual Nodes Energy

**Figure 7:**
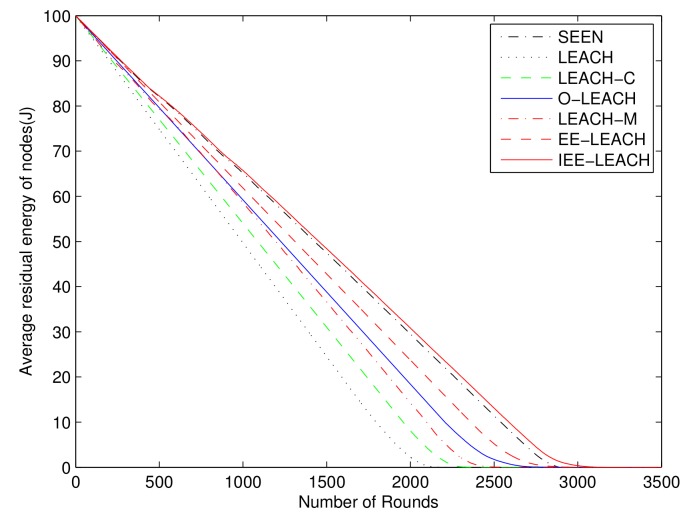
Comparison of residual energy of nodes.

In this test, the effect of the residual nodes energy on the performance is shown. The residual energy of all SNs in the network for the protocols is illustrated in Figure 7. The proposed IEE-LEACH exhibits a slower decrease as the number of rounds increase than a variety of routing protocols. All nodes in the existing LEACH, LEACH-M, O-LEACH, LEACH-C, EE-LEACH, and SEEN protocols are dead after 2267, 2492, 2972, 2422, 3053, and 2889 rounds, respectively. However, all nodes in the proposed IEE-LEACH are dead after 3242 rounds. It can be seen that the new proposed threshold that considers the initial energy of nodes, residual energy of nodes, total energy of the network, and average energy of all nodes increases the network lifetime by about 6% to 43% compared with other routing protocols. Therefore, the strategy presented by the proposed IEE-LEACH protocol can effectively reduce the dissipated energy and extend the lifetime of the network.

### 4.3. The Amount of Transmitted Data

**Figure 8:**
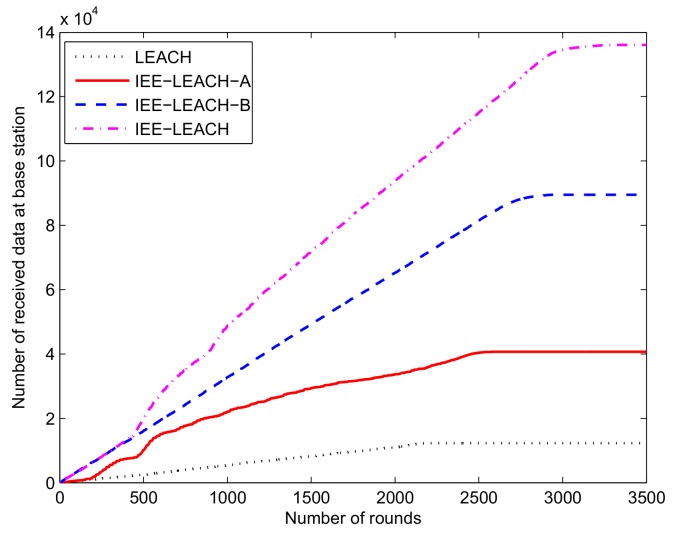
Comparison of data transmission.

In this experiment, we employed the same data aggregation technique and the same number of information bytes. The amount of transmitted data is determined by the number of alive nodes in the entire network, i.e., it is related to the life cycle of nodes. In this section, several tests are performed to evaluate the proposed protocol under different conditions, as displayed in Table 2. Figure 8 depicts the throughput of protocols against the simulation rounds. Since the nodes closer to the BS do not participate in cluster formation and the energy adjustment parameter is used in setting the threshold, it is obvious that the proposed IEE-LEACH protocol is superior to other protocols. Considering that the nodes closer to the BS do not participate in cluster formation, the IEE-LEACH-A improves the throughput of the network; thus, the information transmission performance of the IEE-LEACH-A protocol is better than that of the LEACH protocol. Moreover, the network throughput of the IEE-LEACH-B is larger than that of the IEE-LEACH-A, which illustrates that, in terms of improving the network throughput, the improvement by considering the energy adjustment parameter when setting the threshold is more energy-efficient than when considering the nodes closer to BS which do not participate in cluster formation.

Compared with the LEACH protocol, the IEE-LEACH increases throughput by nearly a factor of 7. Specifically, this method greatly improves the information transmission performance of the network. In addition, compared with the IEE-LEACH-B, the IEE-LEACH has an approximately 30% increase in network throughput. In terms of the amount of transmitted data, the proposed IEE-LEACH protocol sends much more data to the BS in the same round than other protocols.

### 4.4. Comparison of the Average Energy Mean Square Deviation of Nodes

**Figure 9:**
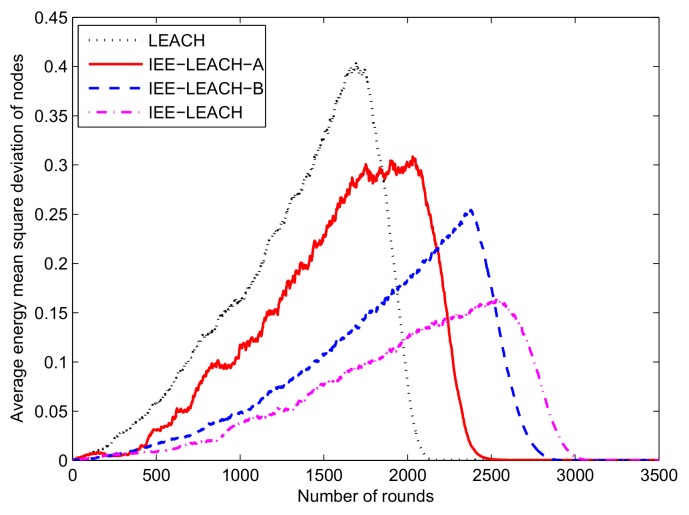
Comparison of average energy mean square deviation of nodes every 10 rounds.

After 10 rounds, we calculate the average energy mean square deviation of the remaining nodes to analyze the energy distribution in the whole network. We can obtain the optimum number of CHs depending on the analysis of the average energy mean square deviation of nodes to transmit information and increase the performance of the entire network. Figure 9 shows that the slope of the LEACH is larger than the proposed protocol before the degree of energy dispersion increases to the maximum. However, the volatility of the proposed IEE-LEACH is less than 0.1. This finding implies that the performance of the proposed IEE-LEACH protocol is better than other protocols in terms of the energy consumption. This improvement is because the proposed IEE-LEACH protocol extends the lifetime of the network by balancing the node energy.

### 4.5. Comparison of the Number of Cluster Heads

To demonstrate that it is necessary to consider the improvement when nodes closer to the BS do not participate in clustering formation, the simulation results of the cluster heads number for the IEE-LEACH-B and IEE-LEACH protocols are provided in this section. Unlike the LEACH and IEE-LEACH-A protocols, IEE-LEACH-B and IEE-LEACH consider the energy when selecting the CH. Thus, IEE-LEACH-B and IEE-LEACH can select the optimal number of cluster heads.

**Figure 10:**
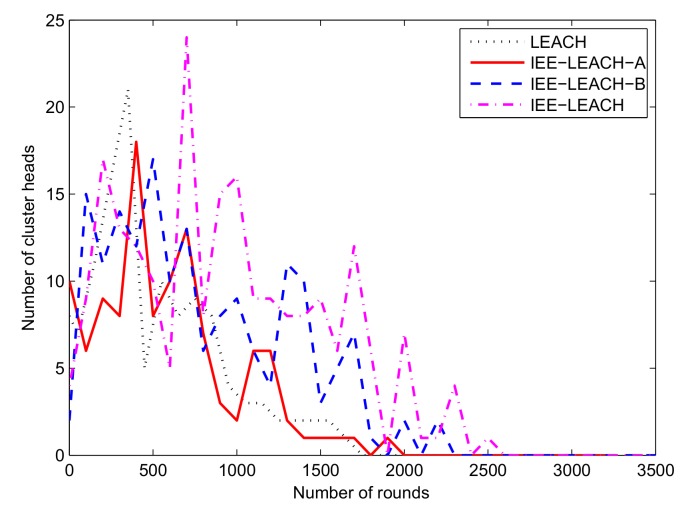
Comparison of the number of cluster heads.

Figure 10 shows the number of cluster heads versus the number of rounds. The maximum number of cluster heads for IEE-LEACH occurs after approximately 700 rounds, and the number of cluster heads is zero after 2600 rounds. However, the maximum number of cluster heads for IEE-LEACH-B occurs in approximately 500 rounds, and the number of cluster heads is zero after 2300 rounds. We find that the number of cluster heads extends up to 2600 rounds for IEE-LEACH as compared to 2300 rounds in IEE-LEACH-B, which is due to the improvement by which the nodes closer to BS do not participate in the cluster formation. The number of cluster heads for IEE-LEACH-A and LEACH disappears after 2050 rounds and 1800 rounds, respectively. It is obvious that IEE-LEACH and IEE-LEACH-B prolong the network lifetime compared with IEE-LEACH-A and LEACH because IEE-LEACH-B and IEE-LEACH consider the energy adjustment parameter when selecting the CH. In addition, the proposed IEE-LEACH protocol generates more CHs than IEE-LEACH-B throughout the 3500 simulation rounds, which can reduce the energy consumption of the WSN nodes. Thus, the proposed scheme is efficient in terms of saving energy and extending the lifetime of the WSN.

### 4.6. Comparison of Total Energy Consumption of the Network

**Figure 11:**
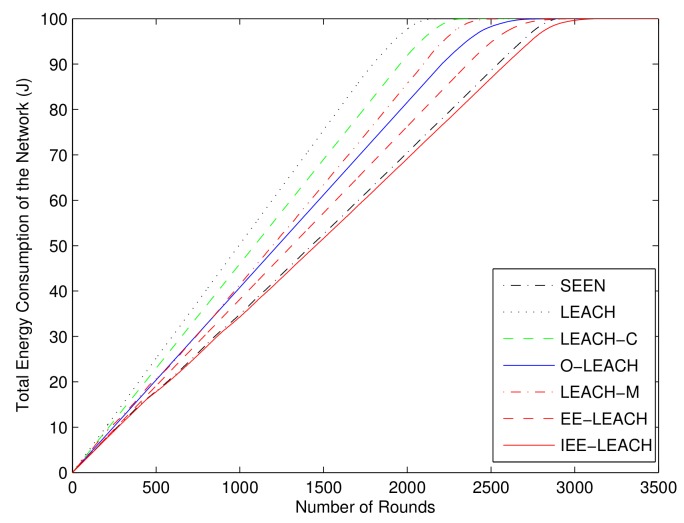
Comparison of total energy consumption of the network.

To prove that the performance of the proposed protocol is better than that of the existing protocols, the total energy consumption of the proposed protocol is compared with that of LEACH, LEACH-M, O-LEACH, LEACH-C [18], EE-LEACH [36] and SEEN, under the same simulation conditions. Figure 11 shows the total network energy consumption of the protocols versus the number of rounds. As shown in Figure 11, the LEACH-C protocol optimizes the cluster head selection and consumes less network energy than the LEACH protocol in the same round. The LEACH-C prolongs the network lifetime to 2723 rounds as compared to 2267 rounds of the LEACH protocol. In addition, the LEACH-M and O-LEACH prolong the network lifetime to 2775 and 2972 rounds, respectively. Because the residual energy of nodes is taken into account when selecting cluster heads and the multi-hop communication mode is applied in EE-LEACH, the EE-LEACH further prolongs the network lifetime to 3114 rounds. The proposed IEE-LEACH protocol not only considers the residual energy and initial energy of nodes when selecting cluster heads, but also considers the average energy of all nodes and the total energy of network to further optimize cluster head selection. Thus, the total network energy consumption of the IEE-LEACH protocol is less than that of the other protocols in the same round, and the proposed IEE-LEACH protocol prolongs the network lifetime to 3242 rounds. Moreover, because single hop, multi-hop and hybrid communication modes are adopted, and the nodes closer to BS than to the CH do not participate in cluster formation, the proposed IEE-LEACH protocol further extends the lifetime of the network.

### 4.7. Performance for Large Sensor Field

**Figure 12:**
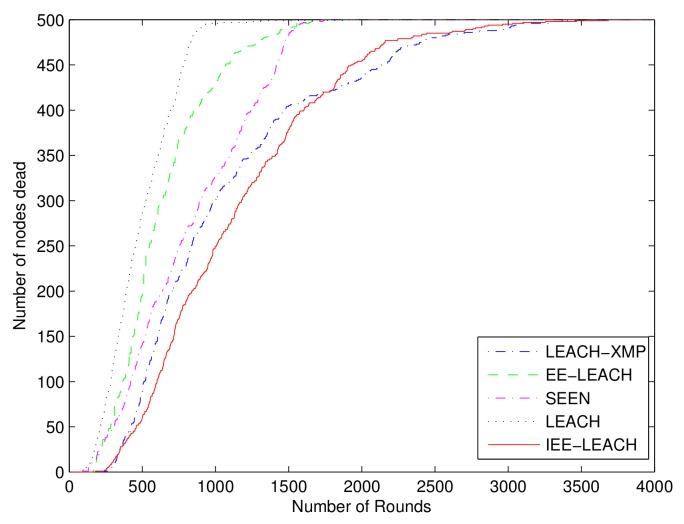
Comparison of life cycles for a large sensor field.

**Figure 13:**
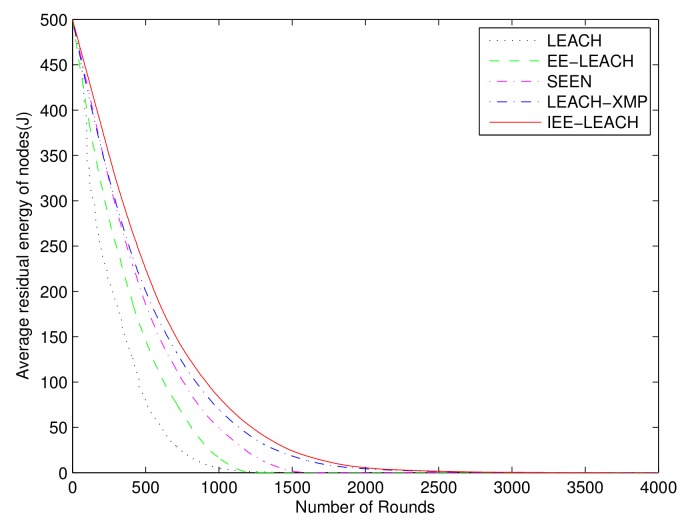
Comparison of residual energy of nodes for a large sensor field.

**Figure 14:**
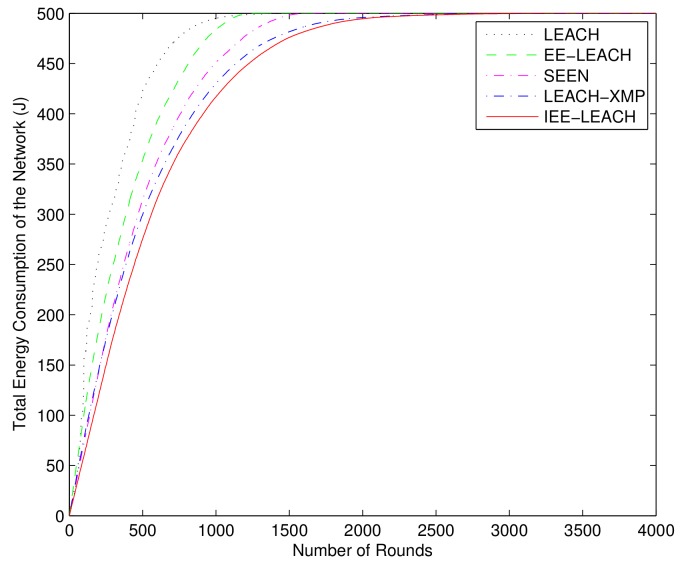
Comparison of total energy consumption of the network for a large sensor field.

To further demonstrate that the performance of the proposed IEE-LEACH protocol is still prominent in large scale, we construct a WSN with randomly deployed 500 sensor nodes in a 500m×500m sensor field. It is clearly from Figure 12 that, compared with the existing routing protocols, the network lifetime is enhanced by the proposed IEE-LEACH protocol as the number of rounds increases. Figure 13 and Figure 14 display the chart view of residual energy and total energy consumption in the network. All nodes in the existing LEACH, EE-LEACH, SEEN and LEACH-XMP protocols are dead after 1328, 1534, 1731 and 2724 rounds, respectively. However, all nodes in the proposed IEE-LEACH are dead after 2692 rounds. It can be seen that the new proposed scheme which considers the initial energy of nodes, residual energy of nodes, total energy of the network, and average energy of all nodes increases the network lifetime by about 5% to 75% compared with different routing protocols. Therefore, it is apparently observed that the proposed IEE-LEACH algorithm performs equally well in case of large network size and is scalable enough for large network areas.

As indicated in Table 3 and Table 4, among the different routing protocols, our protocol can achieve longer network lifetime and lower energy consumption than the others in various scale sensor fields, which is of great significance for diverse practical applications.

## 5. Conclusions

In this paper, a novel clustering protocol, named IEE-LEACH, is proposed to reduce energy consumption and improve the lifetime of WSNs. Compared with the existing routing protocols, the threshold of the proposed IEE-LEACH protocol introduces four parameters: the initial energy of nodes, residual energy of nodes, total energy of the network and average energy of all nodes. This mechanism can improve the robustness of the network and extend the network lifetime. In addition, the proposed protocol can optimize the number of CHs and their distributions, which can effectively reduce the energy consumption. Furthermore, to decrease the energy consumption, we consider that the nodes closer to the BS do not participate in cluster formation. Moreover, the proposed protocol employs single hop, multi-hop, and hybrid communications instead of a single communication mode in data transmission. Therefore, the proposed approach decreases the overall communication cost and significantly improves the network lifetime. The simulation results demonstrate that the proposed IEE-LEACH protocol has a better energy consumption distribution and is more reliable and energy-efficient than some existing protocols.

## Figures and Tables

**Table 1:** Simulation parameters.

Parameters	Values
$E_{elec}$	50 nJ/bit
$E_{DA}$	5 nJ/bit/signal
Transmitter Amplifier $\left(\varepsilon_{\mathrm{fs}}\right)$ if $d\leq d_{0}$	10 pJ/bit/m${}^{2}$
Transmitter Amplifier $\left(\varepsilon_{\mathrm{mp}}\right)$ if $d\geq d_{0}$	0.0013 pJ/bit/m${}^{4}$
*p*	0.05
$d_{0}$	87 m
Data Packet Size	4000 bits
Data Packet rate	1 packet/s

**Table 2:** Comparison of the improvement schemes of the proposed routing protocols.

Protocol Name	Improved Details
LEACH	NULL
IEE-LEACH	Threshold setting considering the energy adjustment parameter, and closer nodes to the BS do not participate in cluster formation.
IEE-LEACH-A	Threshold setting in the same way as LEACH, but closer nodes to the BS do not participate in cluster formation.
IEE-LEACH-B	Threshold setting considering the energy adjustment parameter, but the cluster formation is the same as that of LEACH.

**Table 3:** A performance comparison between different protocols in a 100m×100m sensor field.

Protocol	Lifetime	Residual Energy	Total Energy Consumption
LEACH	1678	2267	2267
EE-LEACH	2326	3053	3114
SEEN	2416	2889	2990
LEACH-C	1836	2422	2723
O-LEACH	2178	2972	2972
LEACH-M	1933	2492	2775
IEE-LEACH	2636	3242	3242

**Table 4:** A performance comparison between different protocols in a 500m×500m sensor field.

Protocol	Network Lifetime	Residual Energy	Total Energy Consumption
LEACH	1361	1328	1411
EE-LEACH	1719	1534	1589
SEEN	1812	1731	1826
LEACH-XMP	3313	2724	2785
IEE-LEACH	3491	2692	2793

## Abbreviations

The following abbreviations are used in this manuscript:

WSNs	Wireless sensor networks
LEACH	Low-energy adaptive clustering hierarchy
CHs	Cluster heads
IEE-LEACH	Improved energy-efficient LEACH
BS	Base station
SNs	Sensor nodes
SEECH	Scalable energy-efficient clustering hierarchy
LA-MHR	Learning automata-based multilevel heterogeneous routing
LEACH-M	LEACH-Mobile
O-LEACH	Optical LEACH
DFS	Distributed fiber sensors
SEEN	Stable energy efficient network
LEACH-XMP	LEACH-eXtended Message-Passing
TDMA	Time division multiple access
CSMA	Carrier-sense multiple access
LEACH-C	LEACH-Centralized
EE-LEACH	Energy-efficient LEACH
